# Killer Bee Molecules: Antimicrobial Peptides as Effector Molecules to Target Sporogonic Stages of *Plasmodium*

**DOI:** 10.1371/journal.ppat.1003790

**Published:** 2013-11-21

**Authors:** Victoria Carter, Ann Underhill, Ibrahima Baber, Lakamy Sylla, Mounirou Baby, Isabelle Larget-Thiery, Agnès Zettor, Catherine Bourgouin, Ülo Langel, Ingrid Faye, Laszlo Otvos, John D. Wade, Mamadou B. Coulibaly, Sekou F. Traore, Frederic Tripet, Paul Eggleston, Hilary Hurd

**Affiliations:** 1 Centre for Applied Entomology and Parasitology, School of Life Sciences, Keele University, Keele, Staffordshire, United Kingdom; 2 Malaria Research and Training Centre (MRTC), Université des Sciences, des Techniques et des Technologies de Bamako, Bamako, Mali; 3 Centre National de Transfusion Sanguine, Bamako, Mali; 4 Institut Pasteur, Centre for Production and Infection of Anopheles (CEPIA), Parasitology and Mycology Department, Paris, France; 5 Department of Neurochemistry Svante Arrhenius v. 21A, Stockholm University, Stockholm, Sweden; 6 Department of Molecular Bioscience, the Wenner-Gren Institute, Svante Arrhenius v. 20C, Stockholm University, Stockholm, Sweden; 7 Temple University Department of Biology, Philadelphia, Pennsylvania, United States of America; 8 Howard Florey Research Laboratories, Florey Institute for Neuroscience and Mental Health, University of Melbourne, Melbourne, Victoria, Australia; Stanford University, United States of America

## Abstract

A new generation of strategies is evolving that aim to block malaria transmission by employing genetically modified vectors or mosquito pathogens or symbionts that express anti-parasite molecules. Whilst transgenic technologies have advanced rapidly, there is still a paucity of effector molecules with potent anti-malaria activity whose expression does not cause detrimental effects on mosquito fitness. Our objective was to examine a wide range of antimicrobial peptides (AMPs) for their toxic effects on *Plasmodium* and anopheline mosquitoes. Specifically targeting early sporogonic stages, we initially screened AMPs for toxicity against a mosquito cell line and *P. berghei* ookinetes. Promising candidate AMPs were fed to mosquitoes to monitor adverse fitness effects, and their efficacy in blocking rodent malaria infection in *Anopheles stephensi* was assessed. This was followed by tests to determine their activity against *P. falciparum* in *An. gambiae*, initially using laboratory cultures to infect mosquitoes, then culminating in preliminary assays in the field using gametocytes and mosquitoes collected from the same area in Mali, West Africa. From a range of 33 molecules, six AMPs able to block *Plasmodium* development were identified: Anoplin, Duramycin, Mastoparan X, Melittin, TP10 and Vida3. With the exception of Anoplin and Mastoparan X, these AMPs were also toxic to an *An. gambiae* cell line at a concentration of 25 µM. However, when tested in mosquito blood feeds, they did not reduce mosquito longevity or egg production at concentrations of 50 µM. Peptides effective against cultured ookinetes were less effective when tested *in vivo* and differences in efficacy against *P. berghei* and *P. falciparum* were seen. From the range of molecules tested, the majority of effective AMPs were derived from bee/wasp venoms.

## Author Summary

Breaking the complex life cycle of malaria by blocking its development in the mosquito is one area of research being pursued for malaria control. Currently, the mosquito itself, or microbes that live within it, are being genetically modified to provide toxic or lethal outcomes to the parasite. However, this usually comes with a cost to the lifespan and reproductive capabilities of the mosquito, resulting in a strong disadvantage if these modified organisms were to be released in the wild. This work aimed to identify a group of molecules suitable for inclusion in genetic modification strategies, which are toxic to malaria parasites, but have no costly side-effects to the mosquito. Within this group of molecules, toxins from bee and wasp venoms were prominent in their effects on mouse and human parasites. These particular molecules may prove effective in novel malaria control strategies and such venoms may also be a promising source of additional anti-malaria toxins.

## Introduction

In the pursuit of malaria eradication, novel tools are in constant demand due to the lack of an effective vaccine and the emergence of pesticide-resistant insects and drug-resistant parasites [1]. Targeting the weak link in the life cycle, namely transmission between the vector and human host, is an historically valid approach to providing reliable and sustainable control [2]. There have been several advances in the development of strategies to block parasite transmission in the vector, aimed at larvae or adult mosquitoes or the sporogonic stages of the malaria parasite [3]. These are being pursued through the use of natural or genetically modified microbes (reviewed in [4]), or through genetic modification of the mosquito vector itself (e.g. [5], [6]). Whilst pathogenic organisms or modified symbionts may become part of an integrated control strategy, it has been suggested that sustained application is challenging in developing countries [7]. An attractive tool for use in control programs would therefore be the production of a genetically modified vector incapable of transmitting the disease, which propagates itself through wild populations without further intervention [8], [9].

To achieve this, transgenic techniques to generate mosquitoes compromised in their ability to transmit malaria and other pathogens are being developed [10]. Genetic modification of the major Asian vector, *An. stephensi*, has been achieved on many occasions (e.g. [6], [11]), whilst transgenesis of the African malaria vector, *An. gambiae*, remains more of a technical challenge. Nonetheless, progress is being made with transgenesis of *An. gambiae* [12], [13], [14], including the development of mechanisms for driving the desired gene through target populations [15], [16]. However, to date, screening for an array of anti-malarial effector molecules that could be incorporated as transgenes into the genomes of relevant mosquitoes or microbes has attracted less attention, and the choice is currently very limited.

We and others have previously proposed that the ideal time to block malaria development in the mosquito occurs within the first 24 hours post-blood meal [17], [18], [19], [20]. To eliminate parasites in the hostile environment of the mosquito midgut, any effector molecules chosen to be expressed in this location must be highly effective, small, soluble, fast acting and resistant to proteolytic digestion [17]. One source of such molecules is present in the evolutionarily conserved innate immune system, namely antimicrobial peptides (AMPs) [21]. AMPs are diverse molecules; many are cationic and amphiphilic; properties that facilitate interactions with negatively charged parasite surfaces rather than neutrally charged mammalian membranes, such as blood cells or macrophages ingested with the parasites [22].

AMPs are broadly divided into membrane active (pore forming through barrel/stave, carpet or torroidal pore mechanisms) and non-membrane active (having intracellular targets such as DNA or RNA, protein synthesis, folding or enzyme activity; e.g. cell penetrating peptides), as reviewed in [21]). They can be further classified according to toxicity, secondary structure, distinct amino acid predominance, presence of cysteine residues, or families of conserved sequences [23]. However, despite extensive studies on structural and physiochemical AMP parameters (reviewed in [24]), it is still impossible to make predictions from the available data as to what will be effective; making anti-*Plasmodium* peptide selection almost arbitrary.

Many candidate AMP molecules have been tested for their activity against various stages of laboratory cultured *Plasmodium* spp. *in vitro* and several have been tested *in vivo* via infections of laboratory-reared anopheline mosquitoes (e.g. [17], [25]). What we are severely lacking, however, is data on field-collected parasites and field-caught mosquitoes from malaria endemic areas.

Here we report the screening of a range of antimicrobial peptides from a variety of sources, including molecules from different antimicrobial structural classes as well as cell-penetrating peptides. As our previous work had uncovered an inverse relationship between upregulation of the mosquito immune system and fecundity [26], we avoided endogenous mosquito immune peptides, as discussed in [17]. Exogenous sources included toxins from bacteria, invertebrate stings and venoms, amphibian skin secretions, fish mucus and vertebrate AMPs. From designer-molecule sources, we included short arbitrary sequences, peptides based on sequence alignments of other AMPs and modified variants of existing effective peptides. Our objective was to identify peptides with efficient anti-*Plasmodium* activity that did not cause detectable fitness costs to the mosquito vector. From initial laboratory screens, we were able to take candidate peptides through initial tests in Mali, mixing gametocytaemic blood with AMPs and feeding through a membrane to recently colonised *An. gambiae*.

## Results

### Initial *in vitro* screens of antimicrobial peptides for toxicity to a mosquito cell line and anti-*Plasmodium* activity

Our initial screen assessed the ability of each of 33 peptides to cause any detrimental effects to an *An. gambiae* cell line and to kill ookinetes of the rodent malaria *P. berghei* (see Table 1 for list and Table S1 for individual peptide information and source). This facilitated rapid screening of peptides for anti-malaria activity using minimal quantities of parasite and peptide material, whilst alerting us to potential toxicity to insects.

**Table 1:** Antimicrobial peptide used in the current study: origin and size.

Peptide name	Origin	Size	Reference
Alytesin	Amphibian	14 aa	[70]
Anoplin	Wasp	10 aa	[43]
Apamin	Bee	18 aa	[71]
Chex1-Arg20 metabolite	Synthetic	19 aa	[36]
Duramycin	Bacteria	19 aa	[72]
Flagellin 22	Bacteria	22 aa	[73]
Granuliberin R	Amphibian	12 aa	[74]
ILF	Synthetic	13 aa	[45]
Indolicidin	Bovine	13 aa	[75]
KLK	Synthetic	11 aa	[76]
Lactoferricin B	Bovine	11 aa	[77], [78], [79]
Levitide	Amphibian	14 aa	[80]
Magainin II	Amphibian	23 aa	[81]
Mastoparan X	Wasp	14 aa	[82]
Melittin	Bee	26 aa	[38]
P2WN	Synthetic	14 aa	[45]
Parasin I	Catfish	19 aa	[83]
Ranatensin	Amphibian	17 aa	[84]
Scorpine	Scorpion	75 aa	[27]
TAT	HIV-1	11 aa	[85]
Temporin A	Amphibian	13 aa	[86]
Temporin B	Amphibian	13 aa	[86]
TP10	Wasp	21 aa	[35]
TP10 (dimer)	Wasp	44 aa	[35]
Ubiquitin	Unspecified	34 aa	[87]
Uperolein	Amphibian	11 aa	[88]
Val-APO	Synthetic	21 aa	[36], [89]
Vida 1	Synthetic	14 aa	[45]
Vida 2	Synthetic	14 aa	[45]
Vida 3	Synthetic	14 aa	[45]
Vida 3 dimer	Synthetic	32 aa	[45]
Vida 4	Synthetic	14 aa	[45]
WKY	Synthetic	5 aa	[90]

A description of all antimicrobial peptides tested in the current study. AMPs were sourced from a variety of organisms displaying antibacterial, antifungal and/or anti-parasitic properties. Custom peptides were also included.

### The effect of AMPs on *An. gambiae* cells *in vitro*

Our first screen focussed on toxicity to the *An. gambiae* cell line, Sua 4.0, using a concentration of 25 µM of each AMP. Only five peptides demonstrated significant impairment of mosquito cell growth over a 48 h period compared with controls: Duramycin, Melittin, TP10 monomer and dimer and Vida3 dimer (Table 2). Of these, Melittin was the most toxic, causing a considerable reduction in cell numbers after 3 h of incubation, with no cells remaining by 24 h. Duramycin caused a similar, though not so rapid, effect with a decrease in cell numbers of 88% by 48 h whereas the decline in cell numbers in response to Vida3 dimer was 62% by 24 h and this remained relatively stable through to 48 h. TP10 reduced cell numbers initially; however, not all cells were affected at this dose, and the population had resumed growth by 24 h. A different pattern was observed when cells were incubated with TP10 dimer; after an initial reduction in cell numbers, no further loss occurred, but growth was impaired for the entire 48 hr period of the experiment.

**Table 2:** Effects of AMPs on *Anopheles gambiae* Sua 4.0 cell growth.

Peptide	Time in culture	Average number of cells (×10^4^/ml) over 3 replicates		Reduction (compared to control)	Significance
		Peptide	Control		
Duramycin	3 hrs	18	31	42%	p<0.001
	24 hrs	13	48	73%	p<0.001
	48 hrs	9	78	88%	p<0.001
Melittin	3 hrs	5	31	84%	p<0.001
	24 hrs	0	48	100%	p<0.001
	48 hrs	0	79	100%	p<0.001
TP10 dimer	3 hrs	24	30	20%	p<0.001
	24 hrs	25	43	42%	p<0.001
	48 hrs	26	62	58%	p<0.001
TP10	3 hrs	22	32	31%	p<0.001
	24 hrs	40	52	23%	p = 0.001
	48 hrs	78	80	3%	p<0.001
Vida 3 dimer	3 hrs	28	30	7%	p = 0.001
	24 hrs	17	45	62%	p<0.001
	48 hrs	19	69	72%	p = 0.001

Effect of AMPs on growth of *Anopheles gambiae* Sua 4.0 cells. Cells were seeded at a density of 30×10^4^/ml with the addition of 25 µM AMP. Cells in different duplicate wells were counted after 3, 24 and 48 h to assess cell growth. Experiments were repeated three times. Differences between cell numbers in peptide and control wells were assessed using one-way ANOVA.

The viability of mosquito cells remaining after 48 h was also assessed for all 33 peptides. The five peptides that caused a significant reduction in cell numbers also caused reduced viability of the remaining cells (Table 3). With the addition of Melittin, no intact cells remained after 48 h. With the addition of Duramycin or TP10 dimer, although cells were still present, Duramycin reduced viability to 0% and TP10 dimer to 1%. Cell viability remained above 50% after 48 h for Vida3 dimer and TP10 monomer. Although Indolicidin did not reduce cell numbers, cell viability was impaired compared with controls. However, it should be noted that the cell population did not expand as rapidly in these control experiments compared with others.

**Table 3:** Effects of AMPs on *Anopheles gambiae* Sua 4.0 cell viability after 48 hrs.

Peptide	Average viability of cells (3 replicates)		Reduction	Significance
	Peptide	Control		
Duramycin	0%	95%	100%	p<0.001
Indolicidin	61%	96%	36%	p<0.001
Melittin	No remaining cells	95%	N/D	N/D
TP10 dimer	1%	96%	99%	p<0.001
TP10	89%	92%	3%	p = 0.03
Vida 3 dimer	58%	94%	38%	p<0.001

Effect of AMPs on viability of *Anopheles gambiae* Sua 4.0 cells. After 48 h of culture, 100 cells were counted in triplicate wells for each peptide for erythrosin B exclusion. Melittin caused 100% lysis of cells, and therefore viability was not assessed. Differences between viability in peptide and control wells were assessed using one-way ANOVA. N/D indicates not determined.

### The effect of antimicrobial peptides on *P. berghei* ookinetes *in vitro*

We also screened all 33 peptides for toxicity against *P. berghei* ookinetes. Ookinete viability ranged from 89–96% after 30 min in control cultures. Only seven peptides demonstrated significant toxicity to ookinetes at a concentration of 50 µM (Table 4). These were, in order of efficacy, Melittin, TP10 dimer, Vida3 dimer, Anoplin, Duramycin, TP10 monomer and Mastoparan X. TP10 dimer and Melittin were toxic to 100% of the ookinetes at 50 µM, and this could also be achieved by increasing the peptide concentration to 100 µM in the case of Vida3 dimer. At this higher concentration, the peptides Duramycin, Anoplin and Mastoparan X reduced viability to 1%, 2% and 4% respectively. To assess whether some of the less effective peptides could act synergistically to increase toxicity, we combined 25 µM concentrations of certain peptides in our *P. berghei* assay. Synergistic effects were recorded in all cases, producing higher ookinete mortality than 50 µM concentrations of a single peptide (Table 4).

**Table 4:** Effect of AMPs on *P. berghei* ookinetes after 30 minutes.

Peptide	Average viability (3 reps)		Reduction
	Peptide	Control	
**50 µM of single peptide**			
Anoplin	10%	93%	89%
Duramycin	11%	94%	88%
Mastoparan X	41%	96%	57%
Melittin	0%	93%	100%
TP10	26%	89%	71%
TP10 dimer	0%	91%	100%
Vida 3 dimer	4%	93%	96%
**100 µM of single peptide**			
Anoplin	2%	93%	98%
Duramycin	1%	93%	99%
Mastoparan X	4%	94%	96%
Vida 3 dimer	0%	94%	100%
**25 µM (each) of two peptides**			
Anoplin+Mastoparan X	28%	94%	70%
Anoplin+Vida 3 dimer	12%	94%	87%
Duramycin+Anoplin	5%	94%	95%
Duramycin+Mastoparan X	3%	95%	97%
Vida 3 dimer+Duramycin	1%	94%	99%
Vida 3 dimer+Mastoparan X	2%	94%	98%

Peptides were incubated with ookinetes for 30 min to assess their speed of action and efficacy. This table summarizes peptides with significant effects on ookinete viability in these conditions (n = 150 ookinetes per treatment in each replicate). Where 100% mortality was not achieved at 50 µM, peptides were doubled in concentration (100 µM) or added in combination with another peptide to total 50 µM (25 µM of each peptide). All results were significant for Wilcoxon Rank sign test at p<0.009.

### The effect of antimicrobial peptides on mosquito fitness

**Figure 1:**
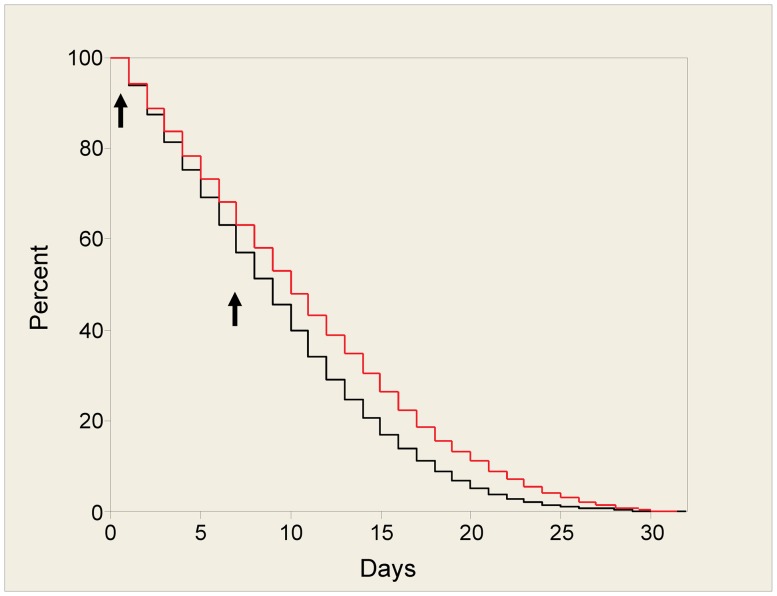
Survival plot for mosquitoes fed with TP10 dimer. Mosquitoes were fed blood containing 50 µM of TP10 dimer, or control on days 0 and 7 (arrows). Fully engorged females from each treatment group were separated into cages of 25 individuals to facilitate counting. Mosquito deaths, from a starting total of 100, were recorded daily. This figure shows the survival plot for one replicate with 50 µM of TP10 dimer, using the Kaplan-Meier method. The black line indicates mosquitoes fed with TP10 dimer, the red line, control mosquitoes.

If incorporated into a transmission blocking strategy, an AMP would be expressed even if the mosquito had not fed on an infective blood meal. In order to assess any effects of ingesting antimicrobial peptides on mosquito fitness, independently from effects caused by malaria infection, we focussed on six of the most promising peptides and screened these candidates by adding them to a bloodmeal to determine any impacts on mosquito longevity and fecundity. At a concentration of 50 µM, none of the peptides had a significant impact on longevity over a 10 day period (Table S2). This period included 2 blood feeds containing AMP, with the analysis weighted to focus on early deaths immediately after the first peptide feed. Each peptide was tested in a minimum of three experiments on different mosquito cohorts. We were able to extend the longevity analysis for three of the peptides (Mastoparan X, TP10 dimer and Vida 3 dimer) to 30 days. Mastoparan X and Vida 3 dimer had no significant long-term effects on longevity. The results for TP10 dimer were inconsistent over four replicate experiments but, overall, it caused a significant reduction in survivorship (p<0.001) (see Figure 1).

The effect of peptides on oviposition was also minimal in these studies. There was no significant effect on the number of eggs laid over two nights for any of the six peptides after the first feed (Table S3). As mosquitoes were kept in cages of 25 individuals, egg numbers were averaged from the number of surviving mosquitoes. According to our method of assessment, fully engorged females laid between 25 and 45 eggs following the first feed. This is far fewer than the number of eggs fully engorged females would be expected to produce. In such artificial laying conditions many eggs may have been retained, with some females not ovipositing. However, this range remained consistent over all replicates for all peptides.

### *In vivo* activity of AMPs against sporogonic stages of *P. berghei* and *P. falciparum*

Promising AMP candidates were assessed for their anti-*Plasmodium* activity and fitness costs *in vivo*, using *P. berghei* (A) and *P. falciparum* from cultured sources (B) and field sources (C).

### A. Antimicrobial peptide activity against *P. berghei*

**Figure 2:**
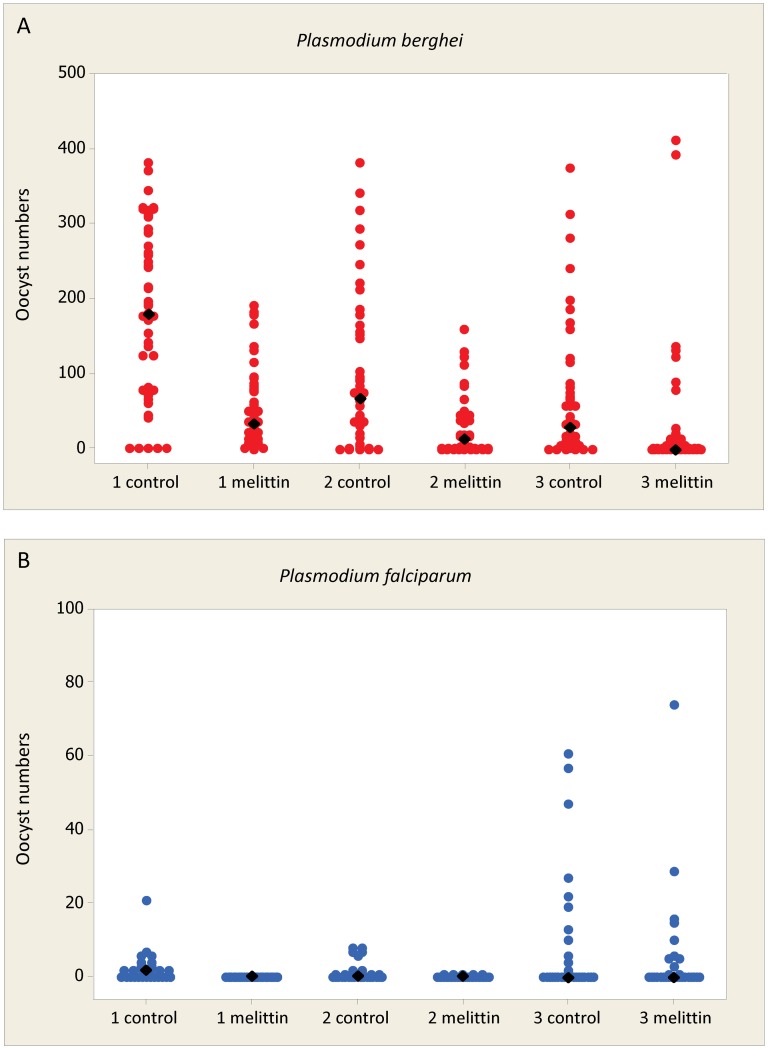
Effect of Melittin on *Plasmodium* development in mosquitoes. Mosquitoes were fed blood containing gametocytes of rodent malaria (fed to *An. stephensi*) or human malaria (fed to *An. gambiae*) supplemented with the AMP Melittin. Fully engorged females were maintained in standardized conditions for 7–8 days prior to dissection for oocyst burdens. Each experiment was performed in triplicate with control feeds containing no AMP. Individual value plots for each dissected midgut are shown. Black diamonds represent the median oocyst burden for each group. Approximately 40–50 individuals were dissected for *P. berghei* infections and 30 individuals for *P. falciparum* infections (see Tables 5 and 6 for full details). A. 50 µM of Melittin added to blood containing *P. berghei* gametocytes. B. 50 µM of Melittin added to blood containing *P. falciparum* gametocytes.

We tested the anti-malaria effects of 50 µM of Anoplin, Duramycin, Melittin, TP10 dimer or Vida3 dimer mixed with mouse blood that contained *P. berghei* gametocytes and fed to *An. stephensi* mosquitoes (Table 5). Mastoparan X and TP10 monomer were omitted from this *in vivo* experiment as they were much less toxic to *P. berghei* ookinetes at a concentration of 50 µM than the other peptides. Mosquito wing sizes did not differ significantly between batches of mosquitoes used in these experiments (p = 0.477) and we therefore make the assumption that, on average, fully engorged females took similar sized bloodmeals and received similar parasite loads. The only consistently effective peptide at this concentration was Melittin, significantly reducing parasite prevalence by an average of 10% (p = 0.019) and intensity by 68% (mean of 37 oocysts per midgut compared to 114 oocysts in control mosquitoes, p<0.001) over the three replicates (Figure 2A). Anoplin (p = 0.930), Duramycin (p = 0.184), TP10 dimer (p = 0.479) and Vida3 dimer (p = 0.953) did not significantly reduce parasite prevalence over three replicate experiments (130–150 mosquitoes dissected per peptide with similar control numbers). In addition, the intensity of infection was not reduced using Anoplin or Vida3 dimer, whilst infection intensity was significantly higher than controls when Duramycin was added (p = 0.013). TP10 dimer reduced intensity (p = 0.039), but not prevalence. Egg production (measured by the number of retained eggs) was not significantly affected by any of the peptides at this concentration. Thus, neither longevity or reproductive fitness are compromised by ingesting these peptides.

**Table 5:** Effect of antimicrobial peptides against *P. berghei* infections in *Anopheles stephensi*.

	Replicate 1		Replicate 2		Replicate 3		
	Peptide	Control	Peptide	Control	Peptide	Control	Significance
**Anoplin**							
Prevalence	66% (44)	73% (45)	98% (50)	96% (50)	100% (50)	97% (35)	N/S
Intensity	21 (0–147)	30 (0–182)	74 (0–580)	179 (0–587)	177 (1–666)	88 (0–344)	N/S
**Duramycin**							
Prevalence	80% (50)	72% (50)	88% (50)	84% (50)	98% (50)	94% (50)	N/S
Intensity	17 (0–114)	11 (0–93)	59 (0–294)	57 (0–285)	135 (0–319)	76 (0–309)	p = 0.013 (▴)
**Melittin**							
Prevalence	98% (50)	100% (50)	82% (49)	90% (49)	72% (50)	88% (50)	p = 0.019 (▾)
Intensity	50 (0–192)	182 (1–371)	28 (0–160)	96 (0–372)	31 (0–412)	66 (0–374)	p<0.001(▾)
**TP10 dimer**							
Prevalence	56% (50)	62% (50)	66% (50)	66% (50)	56% (50)	62% (50)	N/S
Intensity	2 (0–84)	2 (0–87)	4 (0–67)	8 (0–54)	2 (0–13)	2 (0–13)	p = 0.039 (▾)
**Vida 3 dimer**							
Prevalence	33% (40)	34% (50)	80% (50)	92% (50)	76% (50)	68% (50)	N/S
Intensity	1 (0–6)	1 (0–12)	37 (0–271)	47 (0–253)	4 (0–67)	1 (0–7)	N/S

Mosquitoes were provided with a gametocytaemic blood meal mixed with 50 µM of peptide, performed in triplicate. Prevalence (the proportion of infected mosquitoes with total numbers in parentheses) and intensity (mean number of oocysts with the range in parentheses) of infections with paired controls are shown. N/S indicates non-significance. Significant differences are indicated by probability values with (▴) representing oocyst numbers significantly higher than control and (▾) representing oocyst numbers lower than control.

### B. Antimicrobial peptide activity against cultured *P. falciparum*

Melittin, Mastoparan X or TP10 dimer were tested *in vivo* for their effect on the sporogonic stages of *P. falciparum*. Cultured gametocytes were mixed with 50 µM of AMP immediately before feeding to mosquitoes. The anti-*P. falciparum* activity of Vida3, when expressed in tetrameric form in transgenic *An. gambiae* mosquitoes has been reported elsewhere [12] and therefore was not tested here. Mosquito size was again consistent throughout the experiments (p = 0.638). Melittin (Figure 2B) and TP10 dimer were able to reduce prevalence by an average of 60% over three replicates (p<0.001) (Table 6). Both peptides completely blocked infection in one of the replicates. Intensity of infection was reduced by an average of 57% by Melittin (p = 0.001) and 82% by TP10 dimer (p<0.001), although variability between replicate experiments was high. Mastoparan X, which had low anti-*P. berghei* activity, had no effect on *P. falciparum* oocyst prevalence (p = 0.649) or intensity (p = 0.651).

**Table 6:** Effect of antimicrobial peptides against *P. falciparum* cultured gametocytes.

	Replicate 1		Replicate 2		Replicate 3		
	Peptide	Control	Peptide	Control	Peptide	Control	Significance
**Mastoparan X**							
Prevalence	47% (30)	54% (28)	40% (30)	43% (30)	60% (30)	40% (30)	N/S
Intensity	2 (0–11)	2 (0–21)	2 (0–29)	2 (0–8)	11 (0–72)	9 (0–61)	N/S
**Melittin**							
Prevalence	0% (30)	54% (28)	17% (30)	43% (30)	37% (30)	40% (30)	p<0.001
Intensity	0	2 (0–21)	<1 (0–1)	2 (0–8)	6 (0–74)	9 (0–61)	P = 0.001
**TP10 dimer**							
Prevalence	17% (30)	54% (28)	0% (30)	43% (30)	37% (30)	40% (30)	p<0.001
Intensity	<1 (0–2)	2 (0–21)	0	2 (0–8)	2 (0–14)	9 (0–61)	p<0.001

Peptides (final concentration of 50 µM) were mixed with cultured *P. falciparum* gametocytes and membrane-fed to *An. gambiae* mosquitoes (performed at the Pasteur Institute, Paris). For each of three replicates, oocyst prevalence (number of oocyst positive mosquitoes, total number in parentheses) and oocyst intensity (mean number of oocysts present per gut, range in parentheses) were recorded. An equivalent volume of water without peptide was used for the control. N/S indicates non-significance.

### C. Antimicrobial peptide activity against circulating *P. falciparum* from a malaria endemic area

We carried out a preliminary investigation using vectors and parasites from the same malaria endemic district to validate the efficacy of specific peptides in semi-natural conditions. Infecting recently colonised mosquitoes with parasites from gametocyte carriers was extremely challenging, and often resulted in no, or very low, infections. We tested 20 gametocyte carriers, fed to over 8500 mosquitoes; 75% of which took a full blood meal. Wing lengths were consistent throughout the experiments (p = 0.635) and egg numbers were not significantly different in mosquitoes fed with any peptide compared to controls (p = 0.397). Feeds from only nine gametocyte carriers resulted in mosquito infections, with gametocyte numbers ranging from 5–90 gametocytes/µl, producing infections with a prevalence range of 0–46% and intensity of 0–23 oocysts per midgut. Despite this low success rate, we were able to test two of our top candidate AMPs; TP10 dimer (n = 3 replicates) and Vida3 dimer (n = 2 replicates). Over the replicates, TP10 dimer did not provide significant anti-parasitic effects on either prevalence (p = 0.699) or intensity (p = 0.493, Table 7). For Vida3, parasite prevalence was lower than the controls in both replicates. In addition, we were able to carry out single experiments using Mastoparan X, lactoferricin B, levitide and parasin (Table 7). In these preliminary experiments, the first three of these peptides did not reduce parasite prevalence, whilst parasin reduced prevalence by 80%, but did not affect parasite intensity.

**Table 7:** Effect of antimicrobial peptides on field parasites and mosquitoes.

	Replicate 1		Replicate 2		Replicate 3		
	Peptide	Control	Peptide	Control	Peptide	Control	Significance
**TP10 dimer**							
Prevalence	9% (58)	14% (42)	3% (38)	6% (35)	20% (49)	15% (59)	N/S
Intensity	<1 (0–4)	<1 (0–6)	<1 (0–1)	<1 (0–3)	<1 (0–2)	<1 (0–4)	N/S
**Vida 3 dimer**							
Prevalence	3% (63)	14% (42)	0% (45)	6% (35)	N/D	N/D	p = 0.011
Intensity	<1 (0–1)	<1 (0–6)	0	<1 (0–3)			N/S
**Mastoparan X**							
Prevalence	45% (40)	46% (24)	N/D	N/D	N/D	N/D	N/S
Intensity	3 (0–21)	3 (0–23)					N/S
**Lactoferricin B**							
Prevalence	46% (37)	46% (24)	N/D	N/D	N/D	N/D	N/S
Intensity	2 (0–17)	3 (0–23)					N/S
**Levitide**							
Prevalence	8% (60)	15% (59)	N/D	N/D	N/D	N/D	N/S
Intensity	<1 (0–1)	<1 (0–4)					N/S
**Parasin**							
Prevalence	3% (64)	15% (59)	N/D	N/D	N/D	N/D	p = 0.02
Intensity	<1 (0–2)	<1 (0–4)					N/S

Peptides (final concentration of 50 µM) were mixed with human blood containing *P. falciparum* parasites from gametocyte carriers in the village of Nyaganabougou. This was membrane-fed to the progeny of *An. gambiae* mosquitoes collected from neighbouring areas. For each replicate, oocyst prevalence (number of oocyst positive mosquitoes, total number in parentheses) and oocyst intensity (mean number of oocysts present per gut, range in parentheses) were recorded. An equivalent volume of water without peptide was used for the control. N/D indicates not determined. N/S indicates non-significance.

## Discussion

In this study, we concentrated on discovering effector molecules that target the first 24 hours of malaria sporogonic stage development within the mosquito, without affecting mosquito fitness. We tested a large number of effector molecules, the majority of which were chosen due to reported antimicrobial activity (Table S1). Seven of these molecules displayed significant killing effects against malaria parasites, namely Melittin, TP10 monomer and dimer, Vida3 dimer, Anoplin, Mastoparan X and Duramycin. Melittin and TP10 dimer were the most effective anti-ookinete molecules, reducing viability to zero within 30 minutes *in vitro*. For the remaining AMPs, doubling the concentration increased their activity and a synergistic effect was observed when two peptides were combined. In our hands, scorpine was not effective against rodent or human malaria [27] but, perhaps due to its large size, the actual peptide concentration, based purely on the weight of the lyophilized material, may have been lower than expected. The effective molecules were mainly derived from components of bee and wasp venom (except Duramycin and Vida3 dimer). Importantly, none of the peptides had negative impacts on fecundity and only TP10 dimer negatively impacted longevity.

Our seven candidate peptides showed greater efficacy against enriched ookinete cultures than against parasites in the mosquito. As *in vivo* studies encompassed all parasite stages developing within 24 hours (macro- and micro-gametes, zygotes, retorts and ookinetes) these differences may reflect either stage-specific activity or a difference in the experimental environment. If the former, the choice of transgene promoters that direct AMP expression to maturing ookinetes would be critical to maximising the impact of any chosen effector molecule [12]. Promoters also need to be capable of expressing the peptide in sufficient quantities and with the correct temporal and spatial profile. Our findings also support the rationale for including more than one effector molecule in a given transgenic strain, as has been illustrated by other studies. For example, using the Magainin family, a combination of PGLa and Magainin 1 or 2 resulted in a 20 to 50-fold increase in parasite lysis [28], [29].

In parallel, we tested all AMPs for toxicity to an *An. gambiae* cell line, to assess potential impacts on mosquito fitness and help determine speed and mode of action. Of the seven peptides that displayed anti-malarial effects, five also caused a reduction in viability of insect cells. Despite this, feeding AMPs to mosquitoes did not have any significant negative impact on mosquito longevity or fecundity over a 10 day period that included two separate blood meals. These contrasting results may relate to physiological differences between the midgut epithelial cells and cultured cells. The latter lack a glycocalyx, which may act as an initial protective layer in midgut cells [30], enhanced later by the development of the peritrophic matrix [31]. We conclude that the mosquito cell lines currently available are not good models for the midgut epithelium.

One peptide (TP10 dimer) did reduce mosquito longevity in 2 of 4 replicate experiments during the latter stages of a 30 day assessment. However, since no effects were seen prior to 10 days (encompassing at least 2 egg batches) any fitness cost when expressed as a transgene would likely be minimal. This is because the majority of reproductive potential is likely to be realized before this time. To date, investigations of transgenic mosquito fitness indicate variable outcomes. For example, SM1 transgenics showed no fitness cost when fed on uninfected blood [32] and even a fitness advantage when fed on blood infected with *P. berghei* [33]. In contrast, PLA2 transgenics had significantly reduced fitness [32]. We know of no direct evidence that effector molecules used so far have detrimental impacts on fitness *per se*. However, it is possible that perceived fitness costs in transgenic strains may be associated with specific insertion sites, plasmid constructs or loss of genetic heterogeneity as a result of inbreeding [34]. It is therefore important that these potential problems are not compounded and exacerbated by using costly effector molecules.

Some candidate AMPs were additionally investigated using *P. falciparum* and *An. gambiae*. TP10 dimer and Melittin lowered both prevalence and intensity of *P. falciparum* infections produced from laboratory cultures. It has already been reported that the monomer of TP10, added to blood meals at 30 µM, reduced parasite prevalence by 35–45% [35]. Here, in the only direct comparison available with other published data, we show that TP10 dimer at 50 µM concentration reduced prevalence by 35–100%. This again suggests that the ratio of AMP molecules to parasites may be crucial to their effectiveness.

One possible reason for differences *in vivo* and *in vitro* is the midgut environment. Although we have no information on the stability of our AMPs when added to blood, it has been reported that very few peptides survive proteolytic degradation longer than a few minutes [36]. Rapid degradation may not be an issue if small AMPs are delivered over a period of time *via* transgenes under the control of a midgut promoter, or if expressed outside of the midgut to target later stages of the parasite.

Studying AMPs in field conditions in Mali proved challenging, as very few membrane feeds produced infections. Whilst every blood sample drawn was gametocyte-positive, only one sample was able to provide an infection where AMPs were effectively tested. For practical reasons, we conclude that the use of *in vitro*-produced *P. falciparum* gametocytes is the most feasible method for large-scale screening of novel AMPs. What may be best practice, however, is to focus on testing recently isolated parasites from malaria endemic areas, combined with vectors collected from the same area [37].

Whilst we are largely unaware of how these AMPs work in isolation or in synergy, the data presented here provide specific information on our candidate AMPs under given conditions. The Sua 4.0 cell line data confirm the lytic mode of action of Melittin [38]. We can also infer that Duramycin and Vida3 dimer work in a lytic capacity, but at much slower rates at the same concentration and therefore may not be suitable for targeting parasites before they escape into the midgut lumen. TP10 is also toxic to mosquito cells, but without causing cell lysis, supporting the hypothesis that it functions through internal targets rather than membrane lysis [35]. GOR V, a server for predicting the secondary structure of proteins [39], predicts a coil structure for Duramycin and Vida3 dimer whilst confirming that Melittin, TP10 dimer, Mastoparan X and Anoplin are dominated by α-helices, which may be important to their mode of action [40], [41], [42], [43]. It has been possible to distinguish between structural motifs crucial for haemolytic, rather than antibacterial, activity [38] and we may be able to greatly enhance performance of existing AMPs by amino acid substitutions [44], [45], [46]. For instance, D-analogue substitutions for some peptides (including Melittin) have been shown to reduce cytotoxic effects whilst retaining antimicrobial activity [47]. AMPs may also act to enhance the entry of other compounds to the inside of pathogens through transient pores, or hitchhiking with other cell-penetrating peptides.

One benefit of the non-specific nature of AMP activity may be the slow emergence of resistance. Parasites would require profound changes in membrane structure to develop resistance to this class of molecules. Yet, resistance must always be considered as a possibility, and Peschel and Sahl present several mechanisms of how this could evolve [47]. However, it is heartening to know that AMPs have been effective against bacterial infections for at least 100 million years [21].

We have concentrated here specifically on the effects of AMPs on malaria parasites. In the natural environment, AMPs may come into contact with other microbes in both an advantageous or negative manner. On the positive side, AMPs active against malaria may also have activity against other human pathogens such as *Trypanosoma* and *Leishmania* spp. [48], [49]. For example, TP10 was previously shown to have activity against both *P. falciparum* and *Trypanosoma brucei brucei* [35]. This suggests the possibility that transgenic vectors may be engineered for resistance to more than one pathogen. On the negative side, AMPs may be toxic to natural gut microbes that themselves have adverse effects on *Plasmodium* [50]. It must therefore be emphasised that, as an integral part of the mosquito midgut, bacterial flora must be analysed in response to any anti-parasitic molecule deployment.

There is clearly a need to consider how chosen effector molecules might best be deployed through transgenic technologies. Both transgenic and paratransgenic strategies are considered by Wang and Jacobs-Lorena in a recent review [18]. Their deployment in transgenic mosquitoes allow for regulation of time, quantity and localization of effector molecules to best target malaria parasites at specific bottlenecks of development in the mosquito. However, *An. gambiae* is still a challenging species for transgenesis and logistical difficulties in transgene dispersal and reproductive barriers must be overcome [51]. An alternative strategy may be paratransgenesis [52]. Recent advances with engineered natural symbionts from anopheline midguts have shown rapid proliferation of bacteria and subsequent secretion of the desired effector molecule after a blood meal [53] and progress is also being made using the endosymbiont *Wolbachia* [54]. However, paratransgenic approaches also face significant difficulties, including the diversity and stability of infections [55] and fitness costs [56]. Whatever strategy is adopted, the choice of effector molecules remains critical. From the data presented here, AMPs isolated from wasp/bee venoms may provide a class of peptides with potent anti-*Plasmodium* activity that deserves further exploration. If used in synergy and in multiple mosquito compartments, these should be able to eliminate parasites within the vector or be used as blueprints to rationally develop new synthetic AMPs. [57].

In conclusion, we studied a broad range of AMPs and identified Melittin, TP10, Vida3, Mastoparan X and Anoplin as promising candidates to limit malaria transmission in *An. gambiae*. We further suggest that the concentration of the effector molecule, in relation to parasite load, is an important determinant of success. Thus, multiple peptides, acting in synergy, could perhaps achieve a complete transmission blockade within the mosquito. We highlight AMPs from the venom of bees and wasps as a future source of novel anti-*Plasmodium* effector molecules. Over the last few years, bee, wasp and hornet venoms have attracted attention as potential bioactive substances, with new AMPs regularly being described (e.g. [58], [59]).

## Materials and Methods

### Ethics statement

For human participants in Mali: The project was approved by the IRB of the Faculty of Medicine, Pharmacy and Dentistry at the University of Bamako in Mali (Ethical Review N°03/FMPOS). At the study site in Nyaganabougou, ethical approval from community leaders and written informed consent from parents of minors was obtained for all blood samples. Every effort was made to minimise distress and all parasite-positive individuals identified by the study were subsequently treated by trained medical staff.

For animal work at Keele: Animals were housed in the Central Animal Facility at Keele University, which is designated by the Home Office. All work was carried out in accordance with the UK Animals (Scientific Procedures) Act 1986 as amended by EU Directive (2010/63/EU). Protocols were approved by veterinary staff and conducted by trained personnel under Project Licence PPL 40/2997. Every effort was made to minimize suffering.

For animal work in Paris: Animals were housed in the Pasteur Institute Animal Facility, which is accredited by the French Ministry of Agriculture (Accreditation A75-15-31). All work was conducted in accordance with French and European regulations on care and protection of the Laboratory Animals (EC Directive 86/609, French Law 2001-486). Protocols were approved by veterinary staff and performed in compliance with the NIH Animal Welfare Insurance #A5476-01 issued on 31/07/2012. Every effort was made to minimise suffering.

All reagents were purchased from Sigma UK unless otherwise stated. Experiments were conducted at the University of Keele, UK, the Malaria Research and Training Centre (MRTC) at the University of Bamako, Mali and CEPIA at the Pasteur Institute, Paris, France.

### Mosquitoes

Mosquitoes were maintained in standardized conditions [60]. In Keele and Bamako, larval stages were initially reared on Liquifry, followed by TetraMin fish flakes (Tetra, UK). Adults were fed *ad libitum* on 10% glucose supplemented with 0.05% PABA (para-amino-benzoic acid) and females provided with defibrinated horse blood (TCS, UK) for egg production in the UK, O+ human blood in Bamako. In Paris, larvae were fed dry cat food pellets, adults were fed 10% sucrose without PABA and the colony maintained by feeding on anaesthetized rabbits. For *P. falciparum* infections, gametocytes were mixed with AB+ human blood/serum and infected mosquitoes maintained on 10% sucrose supplemented with 0.05% PABA. Wing lengths of experimental mosquitoes were recorded throughout to check for any differences in mosquito size, as differences would affect bloodmeal volume, egg production and numbers of parasites ingested.

The progeny of field-caught mosquitoes from the environs of Kenieroba, Mali (12.6458°N, 7.99222°W) were established as laboratory colonies in Bamako and Keele and were verified as *An. gambiae*, M form, according to the method of Fanello [61]. These mosquitoes were named the Mali strain and were colonized for approximately 15 generations before experiments began to ensure effective membrane feeding rates. The mosquitoes were then used for longevity and fecundity studies at Keele and for *Plasmodium falciparum* infection in Mali for a further 20–30 generations. *An. stephensi* mosquitoes (SDA 500 [62]), the best experimental vector for *Plasmodium berghei* infections, were reared at Keele using the same protocols [63]. *An. gambiae* (Ngousso strain [64], [65]) were used for cultured *P. falciparum* infections in Paris.

### Peptides

A total of 33 peptides were rehydrated in sterile distilled water to a stock concentration of 1 mM and stored at −20°C in aliquots suitable for individual experiments. All peptides were used in assays at a final concentration of 50 µM, unless otherwise stated. This choice of concentration was based on reported stimulated endogenous insect AMP concentrations ranging from 1–100 µM, [66]).

### The effect of antimicrobial peptides on a mosquito cell line

To establish potential effects of AMPs on mosquito cells, the *An. gambiae* Sua 4.0 cell line was used [67]. This facilitated an initial high-throughput screening and provided insights into AMP toxicity, mode of action (lytic or other) and speed of action. Sua 4.0 cells were maintained at 27°C in Schneider's insect medium supplemented with 10% FBS (foetal bovine serum) in T25 culture flasks (Fisher, UK).

Each AMP was tested separately to establish if it affected cell viability and growth. Sua 4.0 cells were seeded in 48 well microtitre plates (BD Biosciences, UK) at a density of 30×10^4^/ml in 195 µl of supplemented Schneider's insect medium, with the addition of 5 µl (final concentration 50 µM) of the test peptide. Cells were maintained for 3, 6, 24 or 48 h, after which they were resuspended and a sub-sample from duplicate wells counted using a haemocytometer. To establish cell viability after 48 h (whether simply arrested in development or non-viable), resuspended cells were added to an equal volume of 5% erythrosin B vital dye and viable cells excluding the stain were counted. The viability of 100 cells in triplicate wells was recorded. Each AMP assay was performed in triplicate and included a negative control minus the peptide (5 µl of water alone added to the medium) and a further control of 5 µl of 10% SDS (sodium dodecyl sulphate) substituted for the AMP as this causes 100% cell lysis.

### The effect of antimicrobial peptides on mosquito longevity and fecundity

Female *An. gambiae* (Mali strain, 3–5 days old) were used to establish the effects of AMPs on mosquito longevity and fecundity in the absence of confounding factors introduced with parasite infection [26]. Two hundred 3–6 day old females were fed on human O+ whole blood supplemented with 50 µM AMP (or water control, 1 ml total volume) via feeding chambers of a Hemotek membrane feeding system. Fully engorged mosquitoes were randomly moved into four separate cages, each containing 25 mosquitoes, to facilitate monitoring. Mortality rates were recorded daily for a 10-day period, and oviposition sites were provided on the second and third night post-feeding. A second blood feed containing the same AMP was administered 7 days later to mimic the likelihood that mosquitoes would feed at least twice in the field during the development of sporogonic stages of the malaria parasite. Oviposition sites were again provided to assess fecundity through the number of eggs laid as an average per surviving mosquito. Assays for each AMP were repeated in triplicate on separate generations of mosquitoes. Due to egg retention under experimental conditions, egg production is best measured by dissecting mosquitoes that have not been provided with an oviposition site. However, this is not a feasible method of determining fecundity when longevity is also being measured.

### The effect of antimicrobial peptides on *Plasmodium berghei* ookinetes *in vitro*

Our primary aim was to target parasites in mosquitoes before they cross the midgut wall, therefore AMPs were first assessed for their ability to kill ookinetes *in vitro*. As protocols for the conversion of *P. falciparum* gametocytes to ookinetes do not provide high yields, the rodent malaria parasite, *P. berghei* ANKA, was used to assess the effects of AMPs on early sporogonic stages. Ookinete cultures of *P. berghei* were established from gametocytaemic blood as previously described [68]. Parasites were enriched using cold 0.17M ammonium chloride after 18 h of culture and washed thoroughly with PBS (phosphate buffered saline). Parasites were seeded in triplicate wells of a 96-well microtitre plate at a density of 1×10^5^/ml in a volume of 47.5 µl Schneider's insect medium (Invitrogen) with 2.5 µl of the test AMP (final concentration of 50 µM). Ookinetes were incubated for 30 min in conjunction with triplicate control wells containing medium with 2.5 µl of water instead of AMP. A sub-sample of parasites was then added to an equal volume of 5% erythrosin B to assess viability (150 ookinetes in total, taken from 3 wells). Each of the 33 AMPs was screened in this way and assays were performed 3 times. AMPs displaying a high degree of toxicity (>90% of ookinetes displaying erythrosin B staining) underwent further ookinete assays using a concentration of 100 µM AMP. To assess synergistic effects, combinations of two peptides were also tested at a concentration of 25 µM per AMP, in the assay described above.

### The effect of antimicrobial peptides on *Plasmodium berghei* sporogonic development *in vivo*

Five of the seven most effective peptides against *P. berghei* ookinetes *in vitro* were subsequently tested on *P. berghei* parasites *in vivo* (the effects of TP10 monomer and Vida3 have been reported elsewhere [35], [45]). To assess the effects of AMPs on parasites *in vivo*, mouse blood containing *P. berghei* gametocytes was obtained by cardiac puncture and 475 µl was rapidly mixed with 25 µl (50 µM final concentration) of AMP. This mixture was fed to 100 nulliparous 3–5 day old female *An. stephensi*. A further 475 µl of blood from the same infection was mixed with 25 µl of distilled water to act as a control. Mosquitoes were maintained at 19°C for optimum parasite development. Between 30 and 50 mosquitoes were dissected after 10 days. Mosquito wing length, numbers of retained eggs and oocysts were recorded in triplicate experiments carried out on different mosquito cohorts.

### The effect of selected antimicrobial peptides on *Plasmodium falciparum in vitro*

*P. falciparum* NF54 gametocytes were produced through large-scale automated culture at the Pasteur Institute, Paris [64], [69]. Gametocytes were grown in 10 ml RPMI 1640 medium (Invitrogen), supplemented with 25 mM HEPES and L-glutamine, 10% heat-inactivated human serum and hypoxanthine (20 mg/L) under a constant gas supply (5% CO_2_, 1% O_2_, 94% N_2_). Fresh red blood cells (RBCs) were added to obtain a 7% haematocrit. Fourteen days after initiating the culture, gametocyte maturity was assessed on thin Giemsa-stained blood smears, and male gametocyte maturity verified by exflagellation tests. Gametocyte cultures were centrifuged for 5 min at 1500 rpm and the ∼500–600 µl pellet resuspended in fresh RBCs and AB human serum to give a final haematocrit of 40%.

Blood containing mature gametocytes (475 µl) was added to 25 µl of the test AMP (50 µM final concentration). Peptides were replaced by 25 µl of sterile deionized water for each control. The blood/parasite/peptide mixture was placed in a Hemotek membrane feeder, previously warmed to 37°C. For each feed, 70 to 100 nulliparous female *An. gambiae* mosquitoes were left to feed in the dark for 15 min and only fully engorged females were transferred to small cages and provided with 10% sucrose containing 0.05% PABA. After 8 days, 30 midguts were dissected and stained with bromo fluorescein for the detection of oocysts.

### The effect of selected antimicrobial peptides on *Plasmodium falciparum in vivo*

#### Study site

Nyaganabougou, a village approximately 60 km south-west of the Malian capital, Bamako, was used as a site for the source of donors of malaria-infected blood. Due to its proximity to the river Niger, which provides perennial mosquito breeding sites, Nyaganabougou has high numbers of infected individuals and an extended transmission season. Mosquitoes used to establish the *An. gambiae* Mali colony used for this study were originally collected in the village of Kenieroba (a distance of 15 km from Nyaganabougou). Potential male and female gametocyte carriers between the ages of 6–10 were screened by thick smear and parasite species and density determined by local staff, led by Saibou Doumbia.

#### *P. falciparum* parasites

Gametocyte carriers were taken to the Malaria Research and Training Centre at the University of Bamako for blood donation. Approximately 4 ml of blood was extracted from carriers and immediately divided into 3 Hemotek membrane feeders for testing 2 separate AMPs and one control for each carrier. Peptides were added to 950 µl of gametocytaemic blood for a final concentration of 50 µM. All parasite-positive individuals were subsequently treated appropriately for parasites and associated symptoms, as determined by, and carried out by, local health workers in Nyaganabougou.

#### Infections

Approximately 120 adult female *An. gambiae* (Mali, 4–7 days old) were starved overnight, and allowed to feed on the gametocytaemic blood/AMP mix for 30 min. Engorged females were maintained on 10% glucose with 0.05% PABA for 8 days before dissection. No oviposition sites were provided so that fecundity could be measured by determining retained eggs. Mosquito wing length, numbers of retained eggs and oocysts were recorded, and triplicate peptide experiments were carried out on different mosquito cohorts, using blood from different donors. Where possible, a minimum of 50 mosquitoes were dissected for each condition.

### Statistical analyses

Analyses were performed using Minitab 16 statistical software. Data were checked for normality (Anderson-Darling) and replicates were checked for between-experiment variation using one-way ANOVA. Sua 4.0 cell growth and viability assays were also analyzed using one-way ANOVA. The effects of AMPs on *P. berghei* ookinetes were assessed by the Wilcoxon signed rank test and oocyst burdens of both rodent and human malaria were compared via the Mann-Whitney U test. Differences in infection prevalence and mosquito sizes (wing length) used in all *in vivo* studies were assessed using a 2-sample *t*-test. Longevity studies were analyzed by Kaplan-Meier tests, where survival curves were compared using the Wilcoxon test to weight early failures (immediately after the first feed) more heavily. The number of eggs produced per mosquito after the first feed was analyzed by a one-way ANOVA.

## Supporting Information

Table S1 **Peptide sequence, activity and source.**
(DOC)

Table S2 **Effect of AMPs on mosquito longevity over 10 days.**
(DOC)

Table S3 **Effect of AMPs on mosquito oviposition over 10 days.**
(DOC)
