# Supplementary material for: Killer Bee Molecules: Antimicrobial Peptides as Effector Molecules to Target Sporogonic Stages of Plasmodium
Source: PLoS Pathog. 2013 Nov 21;9(11):e1003790. doi: 10.1371/journal.ppat.1003790 (PMC3836994; doi:10.1371/journal.ppat.1003790)
Supplement: Table S1 — Peptide sequence, activity and source. (DOC) [file ppat.1003790.s001.doc]

Table S1. Peptide sequence, activity and source.

| **Peptide name** | **Sequence and description** | **Source (& cat. no.)** |
| --- | --- | --- |
| Alytesin | **EGRLGTQWAVGHLM**. Biologically and structurally similar to amphibian bombesin, a homolog of gastrin-releasing peptide of humans. | GenScript (RP10451) |
| Anoplin | **GLLKRIKTLL**. The peptide isolated from the solitary wasp has a broad spectrum activity and homology to Mastoparan X. | GenScript (RP20258) |
| Apamin | **CNCKAPETALCARRCQQH**. A bee venom polypeptide which blocks Ca^2+^-dependent K^+^ channels. | GenScript (RP11002) |
| Chex1-Arg20 metabolite | **ChexRPDKPRPYLPRPRPPRPV**. Derivative of A3-APO – a proline rich peptide optimized for DnaK binding and membrane penetration – where Chex is 1-amino-cyclohexyl-carboxylic acid. | Custom |
| Duramycin | **CKQSCSFGPFTFVCDGNTK**. A lantibiotic that inhibits a number of metabolic processes in eukaryotic cells. | Sigma (D3168) |
| Flagellin 22 | **QRLSTGSRINSAKDDAAGLQIA**. The most conserved region of bacterial flagellin elicits potent immune responses, likely to act as a toll-like receptor agonist. | GenScript (RP19986) |
| Granuliberin R | **FGFLPIYRRPAS**. Granuliberin is a mast cell degranulating peptide, expressed by the skin glands of *Rana rugosa*. | GenScript (RP10459) |
| ILF | **ILPFKFPFFPFRR**. Indolicidin analogue with tryptophans replaced by phenylalanines. Random secondary structure retaining potent antibacterial activity without haemolytic activity. | Custom |
| Indolicidin | **ILPWKWPWWPWRR**. Cationic AMP isolated from cytoplasmic granules of bovine neutrophils. Exhibits potent activity *in vitro* against bacteria and fungi. | GenScript (RP11242) |
| KLK | **KLKLLLLLKLK**  Based on the flesh fly AMP sapecin. Appears to form numerous channels in bacterial membranes resulting in cell death. | GenScript (RP20089) |
| Lactoferricin B | **RRWQWRMKKLG.** Product of pepsin cleavage of bovine lactoferrin. Lactoferrin inhibits *P. falciparum* multiplication by interfering with parasite acquisition of iron. This AMP reduces infectivity of *Toxoplasma gondii* and *Eimeria stiedai*. | Sigma (L1290) |
| Levitide | **pEGMIGTLTSKRIKQ**. Isolated from skin secretions of *Xenopus laevis*. High sequence homology to xenopsin. | GenScript (RP10463) |
| Magainin II | **GIGKFLHSAKKFGKAFVGEIMNS.** AMP from *Xenopus laevis* skin. Forms pores and induces cell permeabilisation. *An. gambiae* infected with a variety of *Plasmodium* spp. injected with magainin into the midgut haemocoel abolished oocyst development >80%. | GenScript (RP11232) |
| Mastoparan X | **INWKGIAAMAKKLL.** Peptidic toxin from hornet venom, similar to TP10, enhancing the movement of ions and lipids. The alpha-helical conformation is thought to be essential for its inhibitory action. | GenScript (10466) |
| Melittin | **GIGAVLKVLTTGLPALISWIKRKRQQ.** From honey bee venom. This peptide binds calmodulin, activates PLA-2 and inhibits protein kinase C. Strongly haemolytic and liberates histamine. | GenScript (RP10290) |
| P2WN | **KWKKALRALARHLK**. Analogue of the murine antimicrobial peptide perforin. Several amino acid replacements results in a purely helical secondary structure. | Custom |
| Parasin I | **KGRGKQGGKVRAKAKTRSS**. Isolated from the skin mucus of wounded catfish. Strong activity (12-100x as strong as magainin 2) against a wide spectrum of microorganisms, without any haemolytic activity. | GenScript (RP11233) |
| Ranatensin | **SNTALRRYNQWATGHFM**. A mast cell degranulating peptide isolated from Rana pipiens. | GenScript  (RP10476) |
| Scorpine | **GWINEEKIQKKIDERMGNTVLGGMAKAIVHKMAKNEFQCMANMCMLGNCEKHCQTSGEKGYCHGTKCKCGTPLSY**. Isolated from scorpion venom, resembling a cecropin/defensin hybrid. This peptide is toxic to *Plasmodium* gametes and ookinetes and appears to work by blocking ion channels. | Custom |
| TAT | **YGRKKRRQRRR**. This cell penetrating peptide from the human immunodeficiency virus has a nuclear localisation signal. | GenScript (RP12784) |
| Temporin A | **FLPLIGRVLSGIL**. Temporins identified from frog skin secretions preserve biological activity in serum, are non-haemolytic and have leishmanicidal activity by compromising membrane integrity. | Custom |
| Temporin B | **LLPIVGNLLKSLL.** As above | Custom |
| TP10 | **AGYLLGKINLKALAALAKKIL.** TP10, is an analogue of the chimera of galanan and mastoparan X linked by a lysine (TP). It is a cell penetrating peptide causing membrane leakage. Effective at inhibiting *P. falciparum* asexual growth and reducing oocyst prevalence. | Custom |
| TP10 (dimer) | **AGYLLGKINLKALAALAKKILGGAGYLLGKINLKALAALAKKIL.** The dimer of TP10 is linked by GG. | Custom |
| Ubiquitin | **MQIFVKTLTGKTITLEVEPSDTIENVKAKIQDKE.**This peptide, found in all eukaryotic cells, has activity against fungal and bacterial pathogens. | GenScript  (RP20508) |
| Uperolein | **QPDPNAFYGLM.** Belongs to tachykinin family and is a vasodilator isolated from amphibian skin. | GenScript  (RP10478) |
| Val-APO | **VRPDKPRPYLPRPRPPRPVRL.** APO is based on many AMP sequences. This peptide has one change from published APO (N-terminal G to V) and has properties that penetrate bacterial membranes and binds to DnaK. | Custom |
| Vida 1 | **KWKKFKKGIGKLFV.** Designer peptide with predicted secondary structure consisting primarily of helices. | Custom |
| Vida 2 | **KWPKFKKGIPWLFV.** Designer peptide with predicted secondary structure consisting primarily of beta sheets. | Custom |
| Vida 3 | **KFPKFRRGIPFLFV.** Designer peptide with predicted secondary structure containing a mixture of sheets & coils. | Custom |
| Vida 3 dimer | **KFPKFRRGIPFLFVGPSGKFPKFRRGIPFLFV.** Dimer linked by GPSG. | Custom |
| Vida 4 | **IFPKFRRGIPFLFV.** Designer peptide similar to Vida 3, replacing the initial K residue with an I to decrease the overall charge. | Custom |
| WKY | **WKYMV.** Synthetic peptide isolated from mixtures of random hexapeptide sequences. | GenScript (RP20299) |
