# Supplementary material for: Killer Bee Molecules: Antimicrobial Peptides as Effector Molecules to Target Sporogonic Stages of Plasmodium
Source: PLoS Pathog. 2013 Nov 21;9(11):e1003790. doi: 10.1371/journal.ppat.1003790 (PMC3836994; doi:10.1371/journal.ppat.1003790)
Supplement: Table S2 — Effect of AMPs on mosquito longevity over 10 days. (DOC) [file ppat.1003790.s002.doc]

Table S2. Effect of AMPs on mosquito longevity over 10 days.

| **Peptide** | **Replicates** | **Percent alive** | | **Significance** | **Wilcoxon rank sign** |
| --- | --- | --- | --- | --- | --- |
|  |  | **Peptide** | **Control** |  |  |
| Anoplin | 3 | 80% | 84% | N/S | p = 0.984 |
| Duramycin | 3 | 84% | 84% | N/S | p = 0.846 |
| Mastoparan X | 3 | 84% | 84% | N/S | p = 0.569 |
| Melittin | 3 | 84% | 84% | N/S | p = 0.675 |
| TP10 dimer | 4 | 76% | 80% | N/S | p = 0.784 |
| Vida 3 dimer | 3 | 68% | 68% | N/S | p = 0.812 |
