# Supplementary material for: Killer Bee Molecules: Antimicrobial Peptides as Effector Molecules to Target Sporogonic Stages of Plasmodium
Source: PLoS Pathog. 2013 Nov 21;9(11):e1003790. doi: 10.1371/journal.ppat.1003790 (PMC3836994; doi:10.1371/journal.ppat.1003790)
Supplement: Table S3 — Effect of AMPs on mosquito oviposition over 10 days. (DOC) [file ppat.1003790.s003.doc]

Table S3. Effect of AMPs on mosquito oviposition over 10 days.

| **Peptide** | **Replicates** | **Mean no. of eggs laid per mosquito** | | **Significance** | **One-way ANOVA** |
| --- | --- | --- | --- | --- | --- |
|  |  | **Peptide** | **Control** |  |  |
| Anoplin | 3 | 36 | 41 | N/S | p = 0.413 |
| Duramycin | 3 | 39 | 41 | N/S | p = 0.437 |
| Mastoparan X | 3 | 44 | 44 | N/S | p = 0.828 |
| Melittin | 3 | 41 | 41 | N/S | p = 0.988 |
| Vida 3 dimer | 3 | 43 | 45 | N/S | p = 0.921 |
| TP10 dimer | 4 | 25 | 25 | N/S | p = 0.596 |
